# Plasma IGFBP-3 and IGFBP-5 levels are decreased during acute manic episodes in bipolar disorder patients

**DOI:** 10.3389/fphar.2024.1384198

**Published:** 2024-04-24

**Authors:** Carlos Fernández-Pereira, Maria Aránzazu Penedo, Adrián Alonso-Núñez, Tania Rivera-Baltanás, Irene Viéitez, José María Prieto-González, María Isabel Vilariño-Vilariño, José Manuel Olivares, Saida Ortolano, Roberto Carlos Agís-Balboa

**Affiliations:** ^1^ Translational Neuroscience Group, Galicia Sur Health Research Institute (IIS Galicia Sur), Área Sanitaria de Vigo-Hospital Álvaro Cunqueiro, SERGAS-UVIGO, CIBERSAM-ISCIII, Vigo, Spain; ^2^ Neuro Epigenetics Lab, Health Research Institute of Santiago de Compostela (IDIS), Santiago University Hospital Complex, Santiago de Compostela, Spain; ^3^ Rare Disease and Pediatric Medicine Group, Galicia Sur Health Research Institute (IIS Galicia Sur), Servizo Galego de Saúde-Universidade de Vigo (SERGAS-UVIGO), Vigo, Spain; ^4^ Translational Research in Neurological Diseases Group, Health Research Institute of Santiago de Compostela (IDIS), Santiago University Hospital Complex, SERGAS-USC, Santiago de Compostela, Spain; ^5^ Neurology Service, Santiago University Hospital Complex, Santiago de Compostela, Spain; ^6^ Physiotherapy, Medicine and Biomedical Sciences Group, Faculty of Health Sciences, University of A Coruña, A Coruña, Spain

**Keywords:** bipolar disorder, Young Mania Rating Scale, insulin-like growth factor 2, insulin-like growth factor-binding proteins, inflammation, monocyte chemoattractant protein 1, macrophage inflammatory protein 1 beta, tumor necrosis factor alpha

## Abstract

**Introduction:** Bipolar disorder (BD) is a recurrent and disabling psychiatric disorder related to low-grade peripheral inflammation and altered levels of the members of the insulin-like growth factor (IGF) family. The aim of this study was to evaluate the plasma levels of IGF-2, insulin-like growth factor-binding protein 1 (IGFBP-1), IGFBP-3, IGFBP-5, IGFBP-7, and inflammatory markers such as tumor necrosis factor α (TNF-α), monocyte chemoattractant protein 1 (MCP-1), and macrophage inflammatory protein 1β (MIP-1β).

**Methods:** We used the Young Mania Rating Scale (YMRS) to determine the severity of the symptomatology, while proteins were measured by enzyme-linked immunosorbent assay (ELISA). We included 20 patients with BD who suffered a manic episode and 20 controls. Some BD patients (*n* = 10) were evaluated after a period (17 ± 8 days) of pharmacological treatment.

**Results:** No statistical difference was found in IGF-2, IGFBP-1, IGFBP-7, TNF-α, and MIP-1β levels. However, IGFBP-3 and IGFBP-5 levels were found to be statistically decreased in BD patients. Conversely, the MCP-1 level was significantly increased in BD patients, but their levels were normalized after treatment. Intriguingly, only IGFBP-1 levels were significantly decreased after treatment. No significant correlation was found between the YMRS and any of the proteins studied either before or after treatment or between IGF proteins and inflammatory markers.

**Discussion:** To some extent, IGFBP-3 and IGFBP-5 might be further explored as potential indicators of treatment responsiveness or diagnosis biomarkers in BD.

## 1 Introduction

Bipolar disorder (BD) is a chronic mental disorder related to severe incapacitating and debilitating symptomatology that impacts both patients and caretakers (McCormick et al., 2015; Nierenberg et al., 2023) and that affects approximately 1%–2% of the global population (Müller and Leweke, 2016; Goes, 2023). According to the World Health Organization (WHO), in 2019, there were 40 million cases of BD all over the world (WHO, 2022). BD is characterized by the recurrent alternation of depressive and manic episodes with periods of a relatively stable mood named euthymia (Lane and Smith, 2023). During a depressive episode, patients experience different symptoms, such as irritability, a feeling of emptiness or sadness, and a generalized loss of pleasure or interest in their daily activities. On the contrary, manic symptoms may include euphoria, increased energy, quick thoughts, over-increased self-esteem, impulsive and reckless behavior, and a reduced need for sleep (Fagiolini et al., 2015; Bauer, 2022). According to the Diagnostic and Statistical Manual of Mental Disorders, Fifth Edition (DSM-V), there are seven possible diagnoses for BD: BD-I, BD-II, cyclothymic disorder, substance/medication-induced bipolar and related disorder, bipolar and related disorder due to another medical condition, other specified and related disorder, and non-specified bipolar and related disorder (Sekhon and Gupta, 2023). Despite this wide range of possibilities, it can be difficult to differentiate BD from other mental disorders. BD diagnostic categories still rely on clusters of symptoms rather than biological markers (Harrison et al., 2018). Therefore, the evaluation of promising biomarkers could be beneficial not just to differentiate between BD diagnostic categories but to also advance treatment strategies and create a more personalized medicine (Scola and Andreazza, 2014).

In this context, we propose to study some members of the insulin-like growth factor (IGF) signaling system, along with some peripheral inflammatory markers that have been connected to bipolar disorder.

To begin with, it has been questioned for a long time whether BD is related to a low-grade inflammatory state (Sayana et al., 2017). Supporting this hypothesis, these studies normally measure peripheral cytokines (Nascimento and Lafer, 2022), microelements (Siwek et al., 2017a; Chebieb et al., 2023), or oxidative stress markers (Jiménez-Fernández et al., 2021) as an indicator of inflammation. Among them, tumor necrosis factor alpha (TNF-α) is one of the most common cytokines studied, which plays a pivotal role in regulating acute and chronic inflammation in the human body (van Loo and Bertrand, 2023). TNF-α is mainly produced by cells of the innate immune system, like macrophages or natural killer cells, as well as cells of the adaptive immune system, like T cells (Clark et al., 2010). In the central nervous system (CNS), TNF-α regulates homeostatic functions, such as neurogenesis, myelination, blood–brain barrier (BBB) permeability, and synaptic plasticity. However, it can also trigger inflammation and neuronal toxicity at pathological levels (Olmos and Lladó, 2014). A meta-analysis of the available data supports that peripheral TNF-α levels are significantly elevated during manic and depressive episodes but not during euthymia in BD patients (Modabbernia et al., 2013; Munkholm et al., 2018; Rowland et al., 2018; Solmi et al., 2021), suggesting the hypothesis that peripheral inflammation might be dependent on the acute mood state rather than a trait mark in BD. On the other hand, chemokines have received less attention than cytokines in the study of BD (Stuart and Baune, 2014). The classical functions of chemokines are derived from their capacity to attract immune cells to the site of inflammation, but they also play roles in neuromodulation and neurogenesis and might be involved in neurobiological processes regarding mental disorders (Ermakov et al., 2023). In the present study, we evaluated monocyte chemoattractant protein 1 (MCP-1) and macrophage inflammatory protein 1 beta (MIP-1β), which belong to the cysteine–cysteine motif chemokine ligands or CCL subfamily (Laing and Secombes, 2004). A meta-analysis based on three different studies revealed that there was no significant difference in MCP-1 levels between BD patients and controls (Modabbernia et al., 2013); however, shortly after, another meta-analysis of five more studies concluded that MCP-1 levels are altered in BD patients but only during depressive episodes (Misiak et al., 2020). In the case of MIP-1β, recent evidence suggests alterations in the onset of BD since individuals with higher polygenic risk scores showed increased MIP-1β levels (Maj et al., 2020).

In the past, some studies found that neurotrophic factors such as the brain-derived neurotrophic factor (BDNF) or nerve growth factor (NGF) could be limiting the damage exerted by neuroinflammation during mood episodes in BD patients both at glial and neuron levels. Since the brain is a well-protected organ, in the last decades, several studies studied the alterations in the peripheral levels of cytokines and neurotrophic factors to correlate its variations with alterations in the CNS measured by subjective tests as an easy, affordable, and consistent manner of identifying biomarkers (Bauer et al., 2014). In this context, the potential role of the IGF signaling system has previously been considered in the field of psychiatry (Pardo et al., 2019). In brief, the IGF system is mainly composed of three ligands (IGF-1, IGF-2, and insulin), IGF-binding proteins (IGFBP-1–7), and their respective cell surface receptors (IGF-1R, insulin receptor-A, insulin receptor-B, and IGF-2R) (Blyth et al., 2020). Both IGF-1 and IGF-2 bind to IGF-1R, IR-A, and IR-B, which leads to the activation of the intracellular tyrosine kinase domain and autophosphorylation that triggers the activation of the insulin receptor substrate (IRS)-initiated phosphatidylinositol 3-kinase-Akt/mammalian target of rapamycin (mTOR) that ends in metabolic processes, and the Ras–mitogen-activated protein kinase (MAPK) pathway, which leads to cell growth and differentiation outcomes (Forbes et al., 2020). Even though IGF-2R has been considered to act as a scavenger mediator, recent evidence might suggest that IGF-2R is capable of triggering signaling pathways mediating IGF-2-derived neurocognitive and neuroprotective effects (Beletskiy et al., 2021). On the other hand, IGFBPs bind to IGFs but not insulin with high affinity and provide an extracellular mechanism to regulate IGF activity either by preventing IGF binding to their respective receptors or by potentiating their actions (Bach, 2018). Nonetheless, IGFBPs also exert IGF-independent actions (Allard and Duan, 2018) and might be interesting to explore in psychiatry as well. IGF-1 has been the most studied IGF member so far in the context of BD. Two different meta-analyses based on three and five studies concluded that IGF-1 peripheral levels are significantly elevated in BD patients (Tu et al., 2016; Chen M et al., 2020).

Recent studies proved that in an animal model, both IGF-1 and IGF-2 systematic injections not only reduce the levels of inflammation markers, such as MCP-1 or TNF-α, but also improve depressive-like behaviors (Luo et al., 2015; Guo et al., 2023). Moreover, both IGF ligands have been shown to exert protection against cytokine-mediated neuronal death (Suh et al., 2013), as it has been proved in the case of TNF-α (Qin et al., 2018). Nonetheless, to the best of our knowledge, no study has yet evaluated peripheral IGF-2 levels in BD patients. Our group previously reported that the plasma levels of IGF-2 were significantly increased in chronic patients of related psychiatric disorders such as schizophrenia (SZ) (Fernández-Pereira et al., 2022) and major depressive disorder (MDD) (Fernández-Pereira et al., 2023) during either a psychotic or a depressive episode, and those levels were normalized after a period of treatment with antipsychotics or antidepressants, respectively. From our perspective, exploring the peripheral relation of inflammatory markers and IGF proteins could be of interest in BD patients. Herein, we evaluate the plasma levels of proteins belonging to the IGF signaling system (IGF-2, IGFBP-1, IGFBP-3, IGFBP-5, and IGFBP-7) and inflammatory markers (TNF-α, MCP-1, and MIP-1β) in BD patients who suffered from a manic episode and how the levels of these proteins could be related to the treatment response and the Young Mania Rating Scale (YMRS) (Young et al., 1978) that measures manic episode severity.

## 2 Materials and methods

### 2.1 Recruiting patients and controls

We present a cross-sectional and partially longitudinal observational study that began in September 2018 and finished in February 2021. We recruited 20 patients who met the DSM-V criteria for bipolar disorder (BD group, *n* = 20) at the Álvaro Cunqueiro Hospital (Vigo, Spain) when they suffered a manic episode and were hospitalized. We also recruited 20 volunteers without any previous psychiatric diagnosis as controls (C group, *n* = 20). Among the 20 patients diagnosed with bipolar disorder, 10 patients were included in a longitudinal group to evaluate treatment effects. Although the BD patients had been prescribed treatment, when they suffered the manic episode, we could not tell whether they were following the prescribed treatment or not.

That is why BD patients who were categorized as “before treatment” (BD0 group, *n* = 10) and “after treatment” (BD1, group, *n* = 10) were under controlled conditions for a period of 17 ± 8 days with treatment that included the following: mood stabilizers (4/10) such as lithium or valproate acid; different atypical antipsychotics (10/10) such as olanzapine, aripiprazole, quetiapine, risperidone, asenapine, or paliperidone; antidepressants (2/10) like sertraline and venlafaxine; and benzodiazepines (7/10) such as lorazepam, flurazepam, or diazepam. We excluded possible controls who had a history of any psychiatric condition or if they had a viral infection or cardiovascular disease.

The inclusion criteria included meeting the DSM-V bipolar disorder diagnostic criteria, age equal to or above 18 years old (≥18 years), and proper delivery of signed written consent from patients or their authorized legal guardians. The exclusion criteria included additional neurological pathologies or other diseases that could have interfered with our study, such as viral infections, cancer, or cardiovascular diseases. During the selection process, we also excluded women with conditions such as pregnancy or lactation. This research was carried out according to the requirements of the Declaration of Helsinki and approved by the Research Ethics Committee of Pontevedra–Vigo–Ourense (Code 2018/598).

### 2.2 Blood collection and protein measurement

Patients came to the hospital suffering from a manic episode. Blood was extracted immediately and collected in EDTA tubes, and then, plasma and leucocytes were separated in a Ficoll-Paque (3 mL, 1:1 blood) gradient by centrifugation (2,000 rpm, 35 min). Finally, plasma was aliquoted and stored in the freezer (−80°C) until protein measurement.

We defrosted the plasma samples gradually at room temperature. Then, we measured the plasma levels of the proteins belonging to the IGF signaling system, IGF-2 (Catalog N° EH0166), IGFBP-1 (EH0167), IGFBP-3 (EH0169), IGFBP-5 (EH0405), and IGFBP-7 (EH0171), using ELISA commercial kits (Fine Biotech Co., Ltd., Wuhan, China). We also measured the plasma levels of the inflammatory markers, MCP-1 (Catalog N° DYZ279-05), MIP-1β (N° DYZ271-05), and TNF-α (N° DYZ210-05), using DuoSet^®^ ELISA kits (R&D Systems, Bio-Techne). In both cases, we followed the manufacturer’s instructions. We measured all samples at least in duplicate, and the intra-assay coefficient of variation (CV) was below 8% in IGF proteins and 10% in inflammatory markers.

### 2.3 Subjective scale

An expert clinician at the Alvaro Cunqueiro Hospital measured the YMRS (Young et al., 1978) to assess manic symptoms. The YMRS has 11 items and is based on the subjective report of the patient in the last 48 h. Each of the 11 items in the YMRS is given a severity rating. The 11 items are as follows: 1) elevated mood; 2) increased motor activity–energy; 3) sexual interest; 4) sleep; 5) irritability; 6) speech (rate and amount); 7) language–thought disorder; 8) content; 9) disruptive–aggressive behavior; 10) appearance; and 11) insight. Items 1–4, 7, 10, and 11 are given ratings of 0–4, whereas items 5, 6, 8, and 9 are given 0–8 points, which makes a theoretical total of 60 points.

By the time they were hospitalized, the BD patients had a YMRS mean of 29.8 ± 9.40 points, which was reduced to 14.7 ± 3.92 points after treatment, with a mean YMRS percentage reduction of 49.82% ± 6.32%. Since the remission criterion has been normally considered to be at YMRS <12 or <8 points (Tohen et al., 2009), we can speculate that there is a notable recovery in the BD patients from manic symptomatology but not full remission. Nonetheless, the 50% reduction criterion has been considered a measure of treatment responsiveness (Chengappa et al., 2003; Berwaerts et al., 2011).

### 2.4 Statistical analysis

We show quantitative data as the mean and standard deviation (M ± SD) for each parameter. We used the Shapiro–Wilk test to check whether quantitative parameters (age, IGF-2, IGFBP-1, IGFBP-3, IGFBP-5, IGFBP-7, MCP-1, MIP-1β, TNF-α, and YMRS) could be adjusted to a normal distribution (represented as SW (df) = F, *p*-value >0.05) or not (*p* < 0.05). If these parameters were normally distributed and positive for Levene’s test (*p* > 0.05), we used parametric Student’s t-test (t (df) = F, *p*-value) to compare the distributions of each parameter among groups in order to identify significant differences. If not, we used a non-parametric test such as the Mann–Whitney test (U, *p*-value). When comparing longitudinal data, we used a parametric paired *t*-test or a non-parametric paired test such as the Wilcoxon matched-pairs signed-rank test (W, *p*-value). When looking for correlations among parameters, we used Pearson’s correlation coefficient (rp (df) = rp, *p*-value) if both variables followed a normal distribution or Spearman’s correlation coefficient (rs (df) = rs, *p*-value) if at least one distribution did not adjust. In the case of a significant correlation among parameters that could act as confounding variables, we used multiple linear regression analysis with the backpropagation method to predict the effect of different independent variables (condition, age, and gender) on the values of dependent variables (IGF-2, IGFBP-1, IGFBP-3, IGFBP-5, IGFBP-7, MCP-1, MIP-1β, and TNF-α). We used GraphPad Prism version 7.05 (License Serial number: GP7-1098034-R###-#####) to analyze the data.

## 3 Results

### 3.1 Levels of IGF proteins in C and BD patients

**FIGURE 1:**
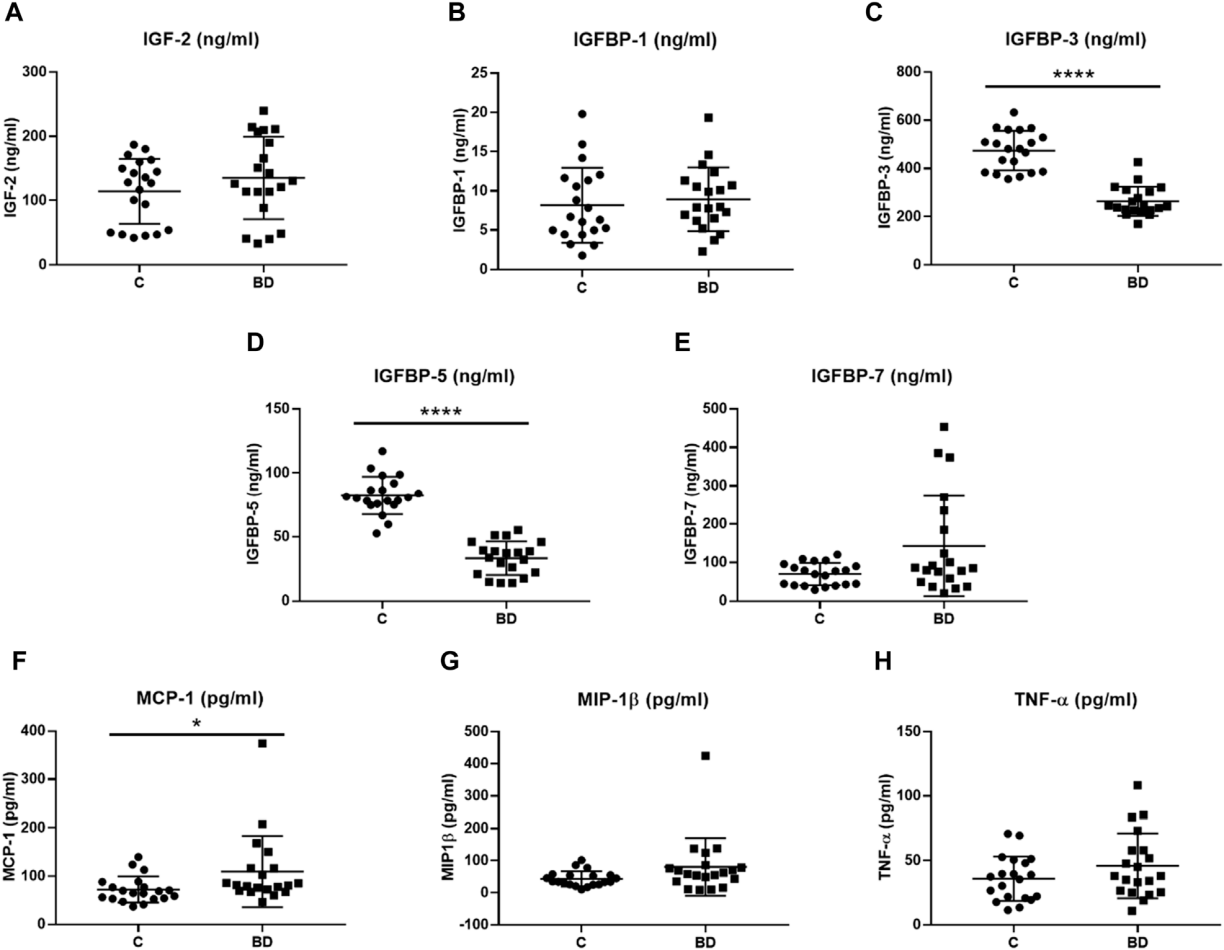
Protein plasma levels of insulin-like growth factor (IGF) proteins (ng/mL) and inflammatory markers (pg/mL) in controls (C, *n* = 20) and bipolar disorder (BD, *n* = 20) patients at hospital admission. **(A)** IGF-2 (ng/ml), **(B)** IGFBP-1 (ng/ml), **(C)** IGFBP-3 (ng/ml), **(D)** IGFBP-5 (ng/ml), **(E)** IGFBP-7 (ng/ml), **(F)** MCP-1 (pg/ml), **(G)** MIP-1β (pg/ml) and **(H)** TNF-α (pg/ml) plasma levels were compared. C, healthy control; BD, bipolar disorder; IGF, insulin-like growth factor; IGFBP, insulin-like growth factor-binding protein; MCP-1, monocyte chemoattractant protein 1; MIP-1β, macrophage inflammatory protein 1 beta; TNF-α, tumor necrosis factor alpha. * *p*-value < 0.05 and **** *p*-value < 0.0001.

We found that the plasma levels of IGFBP-3 (t (38) = 9.24, *p*-value <0.0001) and IGFBP-5 (t (38) = 11.14, *p*-value <0.0001) were significantly decreased in bipolar patients compared to controls (Figures 1C, D; Table 1). However, the plasma levels of IGF-2 (U = 227, *p*-value = 0.478), IGFBP-1 (t (38) = 0.53, *p*-value = 0.597), and IGFBP-7 (U = 252, *p*-value = 0.165) remained non-significant between BD patients and C (Figures 1A, B, E, respectively; Table 1).

**TABLE 1:** Mean values and standard deviations of the proteins in controls (C) and bipolar disorder (BD) patients.

Parameter	C (N = 20)	BD patients (N = 20)	*p*-value
Age (years)	48.80 ± 7.34	51.10 ± 9.85	0.408^a^
Gender (F/M)	10/10	10/10	-
IGF-2 (ng/mL)	114.53 ± 50.59	135.25 ± 64.02	0.478^b^
IGFBP-1 (ng/mL)	8.17 ± 4.78	8.92 ± 4.06	0.597^a^
IGFBP-3 (ng/mL)	474.33 ± 82.24	264.11 ± 60.22	**< 0.0001** ^a^
IGFBP-5 (ng/mL)	82.46 ± 14.77	33.35 ± 13.06	**< 0.0001** ^a^
IGFBP-7 (ng/mL)	70.09 ± 28.89	143.21 ± 130.69	0.165^b^
MCP-1 (pg/mL)	72.31 ± 27.18	109.33 ± 73.92	**< 0.05** ^a^
MIP-1β (pg/mL)	43.59 ± 23.66	80.52 ± 87.42	0.068^a^
TNF-α (pg/mL)	36.01 ± 17.31	46.06 ± 24.64	0.150^a^

The *p-*value was calculated and is reported according to the APA style. The following tests were applied:

^a^ Unpaired Student’s t-test.

^b^ Mann–Whitney test.

C, control group; BD, bipolar disorder; IGF, insulin-like growth factor; IGFBP, insulin-like growth factor-binding protein; MCP-1, monocyte chemoattractant protein 1; MIP-1β, macrophage inflammatory protein 1 beta; TNF-α, tumor necrosis factor alpha.

In all samples, only IGFBP-7 plasma protein levels were significantly and positively correlated with age (r_s_ (40) = 0.319, *p*-value = 0.045; Supplementary Table S1). This correlation between IGFBP-7 and age was still maintained in the control group (r_p_ (20) = 0.506, *p*-value = 0.023; Supplementary Table S1), although it was not present in the BD group (r_s_ (20) = 0.135, *p*-value = 0.570). No other significant correlation was found between any member of the IGF signaling system measured in this study and age (Supplementary Table S1). In the case of gender, we did not find any significant difference between male and female IGF levels in the whole sample. This non-significant difference was maintained both in the control group and BD group of patients (Supplementary Table S2). Bearing this in mind, we further analyzed IGFBP-7 levels between controls and BD patients, correcting for age. We calculated a linear regression model using the backpropagation method to predict the effects of independent variables such as the group (control and bipolar disorder) and age (years) over IGFBP-7 levels as dependent variables. The model excluded the variable age, meaning that the levels of IGFBP-7 can be better explained by the variable group (Table 2).

**TABLE 2:** Linear regression model was performed using the backpropagation method, excluding variables from the model that do not have a significant impact on the variance explained in the levels of IGFBP-7 and MIP-1β.

IGFBP-7 plasma levels (ng/mL)						
Predictors	F (df1, df2)	R	*p-*value	β	SD	*p-*value
*Model 1*	2.93 (2, 39)	0.137	0.066	51.35	89.95	0.572
Group				72.24	30.59	0.024
Age				0.38	1.79	0.831
*Model 2*	5.97 (1, 39)	0.136	0.019	70.09	21.16	0.002
Group				73.12	29.93	0.019
MIP-1β plasma levels (pg/mL)						
*Model 1*	4.02 (2, 39)	0.178	0.026	−78.54	89.95	0.190
Group				31.17	20.01	0.128
Age				2.50	1.17	0.039
*Model 2*	5.41 (1, 39)	0.125	0.026	−75.22	59.90	0.217
Age				2.75	1.18	0.026

Df1, degrees of freedom of the independent variables; df2, degrees of freedom of the dependent variable; R^2^, R-squared; *p*-value, *p*-value of the regression equation of each model; β, beta coefficient; SD, standard deviation of the beta coefficients; *p* = *p*-value of the beta coefficients. The group is a dichotomic variable formed from mental conditions (controls, bipolar disorder). Age is a continuous variable. IGFBP, insulin-like growth factor-binding protein; MIP-1β, macrophage inflammatory protein 1 beta.

### 3.2 Levels of inflammatory cytokines in C and BD patients

We observed that the plasma levels of the inflammatory marker MCP-1 were significantly increased in BD patients (U = 110, *p*-value = 0.0143) compared to controls (Figure 1F; Table 1). The significant difference (U = 110, *p*-value = 0.0243) was still present after removing the outlier (Figure 1F). Nonetheless, the other two inflammatory markers, MIP-1β (U = 132, *p*-value = 0.068) and TNF-α (t (38) = 1.47, *p*-value = 0.150), remained statistically undifferentiated between BD patients and controls (Figures 1G, H; Table 1).

The levels of MIP-1β were significantly correlated with age in the whole sample (r_s_ (40) = 0.316, *p*-value = 0.047). However, this correlation was lost in the control group, but it was maintained in the BD group of patients (r_s_ (20) = 0.470, *p*-value = 0.036). On the other hand, no other correlation was found between MCP-1, MIP-1β, and TNF-α levels and age (Supplementary Table S1). No significant difference was observed in the plasma levels of the inflammatory markers between males and females in any group (Supplementary Table S2). Therefore, we then analyzed and compared the MIP-1β levels between controls and BD patients, correcting for age. In the case of MIP-1β, the model excluded the variable group, meaning that the levels of MIP-1β can be better explained by the variable age (Table 2).

### 3.3 Levels of IGF proteins and inflammatory markers before and after treatment

**FIGURE 2:**
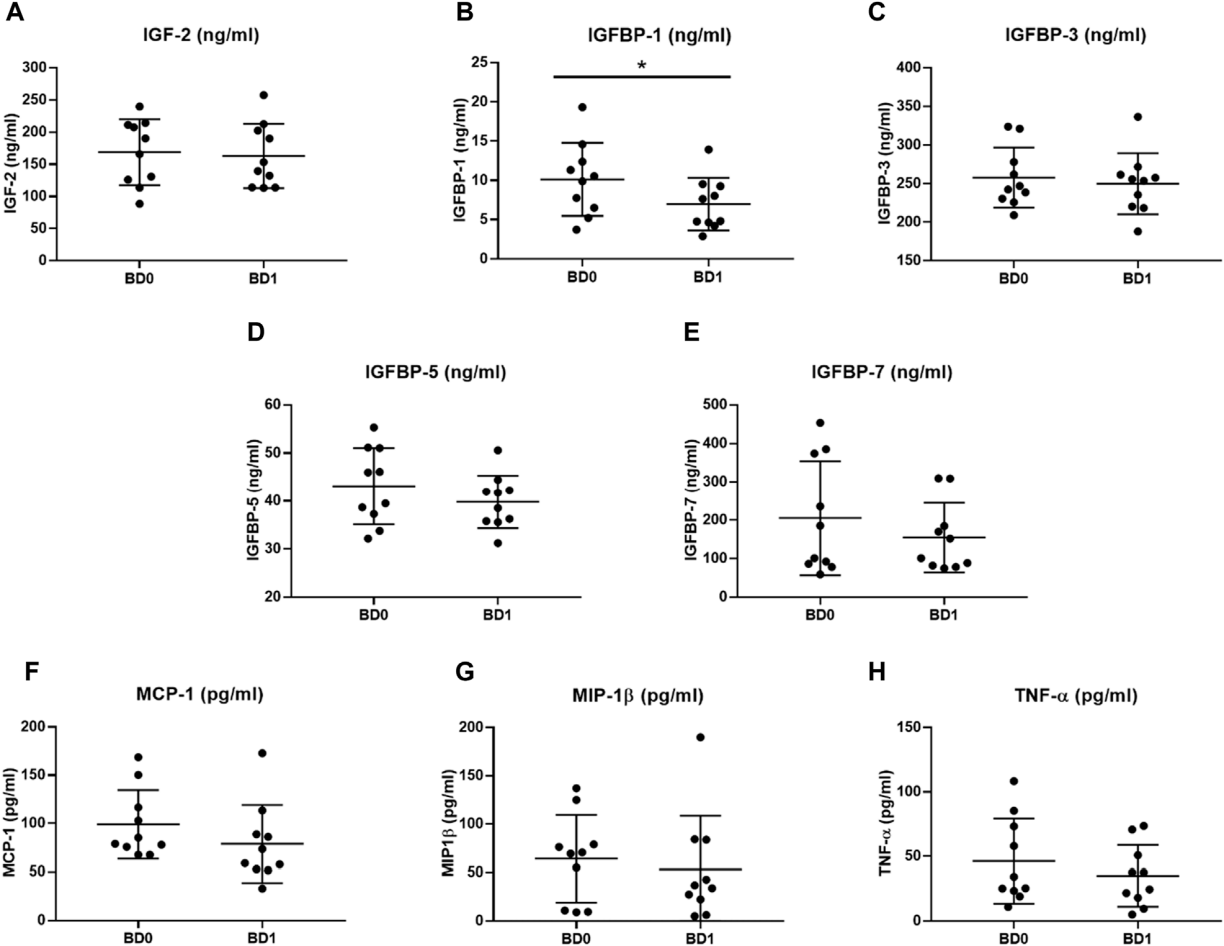
Protein plasma levels of IGF proteins (ng/mL) and inflammatory markers (pg/mL) in BD patients before starting treatment at hospital admission (BD0, *n* = 10) and after treatment at hospital discharge (BD1, *n* = 10). **(A)** IGF-2 (ng/ml), **(B)** IGFBP-1 (ng/ml), **(C)** IGFBP-3 (ng/ml), **(D)** IGFBP-5 (ng/ml), **(E)** IGFBP-7 (ng/ml), **(F)** MCP-1 (pg/ml), **(G)** MIP-1β (pg/ml) and **(H)** TNF-α (pg/ml) plasma levels were compared. C, healthy control; BD, bipolar disorder; IGF, insulin-like growth factor; IGFBP, insulin-like growth factor-binding protein; MCP-1, monocyte chemoattractant protein 1; MIP-1β, macrophage inflammatory protein 1 beta; TNF-α, tumor necrosis factor alpha. * *p*-value < 0.05.

After treatment, the BD1 group had significantly lower levels of IGFBP-1 (W (10) = −53, *p* = 0.0039) (Figure 2B; Table 3). On the other hand, IGF-2 (t (10) = 0.624, *p* = 0.548), IGFBP-3 (t (10) = 0.829, *p* = 0.429), IGFBP-5 (t (10) = 1.496, *p* = 0.169), and IGFBP-7 (W (10) = −9, *p* = 0.652) levels were non-significant after treatment (Figures 2A, C–E; Table 3). In the case of inflammatory markers, the overall tendency was the reduction in their concentrations after treatment (Figure 2; Table 3), but none of them achieved significant differences: MCP-1 (W (10) = −25, *p* = 0.232), MIP-1β (W (10) = −15, *p* = 0.492), and TNF-α (t (10) = 1.653, *p* = 0.133) (Figures 2F–H; Table 3). No correlation was found between age and any protein in the BD0 and BD1 groups (Supplementary Table S1). In the case of gender, the protein levels of men were statistically lower in both IGFBP-3 (female: 286.39 ± 34.76 vs. male: 229.26 ± 13.08, U = 0, *p*-value = 0.008) and IGFBP-5 (female: 48.60 ± 6.08 vs. male: 37.59 ± 5.42, t (8) = 3.025, *p*-value = 0.016) in the group of patients after treatment (Supplementary Table S2).

**TABLE 3:** Mean values and standard deviations of the proteins in bipolar patients before treatment at admission (BD0) and bipolar patients after treatment at hospital discharge (BD1).

Parameter	BD0 (*n* = 10)	BD1 (*n* = 10)	*p*-value
Age (years)	53.10 ± 10.18		-
Gender (F/M)	5/5		-
IGF-2 (ng/mL)	168.91 ± 51.27	162.97 ± 50.06	0.548^a^
IGFBP-1 (ng/mL)	10.12 ± 4.66	6.96 ± 3.35	**< 0.01** ^b^
IGFBP-3 (ng/mL)	257.83 ± 38.98	249.93 ± 39.63	0.429^a^
IGFBP-5 (ng/mL)	43.09 ± 7.94	39.81 ± 5.51	0.169^a^
IGFBP-7 (ng/mL)	205.08 ± 148.79	154.86 ± 90.42	0.652^b^
MCP-1 (pg/mL)	99.14 ± 35.48	78.91 ± 40.20	0.232^b^
MIP-1β (pg/mL)	64.21 ± 45.19	53.27 ± 55.27	0.492^b^
TNF-α (pg/mL)	46.32 ± 32.93	34.93 ± 23.92	0.133^a^
YMRS (points)	29.8 ± 9.40	14.7 ± 3.92	**< 0.0001** ^a^
Treatment	Atypical antipsychotics (10/10)		
	Antidepressants (2/10)		
	Benzodiazepines (7/10)		
	Mood stabilizers (4/10)		
	Lithium (2/4)		
	Valproic acid (2/4)		

The *p-*value was calculated and is reported according to the APA style. The following tests were applied:

^a^ Paired Student’s t-test.

^b^ Wilcoxon matched-pairs signed-rank test.

BD, bipolar disorder; IGF, insulin-like growth factor; IGFBP, insulin-like growth factor-binding protein; MCP-1, monocyte chemoattractant protein 1; MIP-1β, macrophage inflammatory protein 1 beta; TNF-α, tumor necrosis factor alpha. YMRS, Young Mania Rating Scale.

### 3.4 Correlation between IGF proteins and inflammatory markers

The levels of IGF-2 were correlated with IGFBP-7 levels in the overall sample (r_s_ (40) = 0.814, *p*-value <0.0001), i.e., controls (r_s_ (20) = 0.836, *p*-value <0.0001) and bipolar patients (r_s_ (20) = 0.767, *p*-value <0.0001), both before (r_s_ (10) = 0.903, *p*-value <0.0001) and after treatment (r_s_ (10) = 0.737, *p*-value = 0.015) (Supplementary Table S3). No other significant correlation was found between IGF-2 and IGFBP-1, IGFBP-3, or IGFBP-5 levels (Supplementary Table S3). Nonetheless, IGFBP-3 levels were significantly correlated with IGFBP-5 levels in the overall sample (r_s_ (40) = 0.810, *p*-value <0.0001) and the control group (r_p_ (20) = 0.653, *p*-value = 0.002) but not in the BD group (r_p_ (20) = −0.082, *p*-value = 0.732). Curiously, this correlation was present in the BD group both before (r_p_ (20) = 0.953, *p*-value <0.0001) and after treatment (rp (20) = 0.811, *p*-value = 0.004) (Supplementary Table S3). On the other hand, there was a significant correlation between IGFBP-1 and IGFBP-7 levels overall (r_s_ (40) = 0.322, *p*-value = 0.043; Supplementary Table S3). Finally, IGFBP-3 levels were also significantly correlated with IGFBP-7 levels in the control group (r_s_ (20) = 0.459, *p*-value = 0.042) and the group of BD patients but only after treatment (r_s_ (20) = 0.652, *p*-value = 0.041; Supplementary Table S3). In the case of inflammatory markers, TNF-α was not significantly correlated with MCP-1 or MIP-1β. Conversely, MCP-1 and MIP-1β were significantly correlated in the overall group (r_s_ (40) = 0.496, *p*-value = 0.001) and in bipolar patients before treatment (r_s_ (10) = 0.758, *p*-value = 0.011; Supplementary Table S4).

The levels of MCP-1 in the overall group were positively correlated with the levels of IGFBP-1 (r_s_ (40) = 0.463, *p*-value = 0.003) (Table 4). The correlation between MCP-1 and IGFBP-1 was still maintained in the control group (r_s_ (20) = 0.572, *p*-value = 0.008) but lost in bipolar patients (r_s_ (20) = 0.377, *p*-value = 0.101; Table 3). Conversely, both IGFBP-3 (r_s_ (40) = −0.444, *p*-value = 0.004) and IGFBP-5 (r_s_ (40) = −0.314, *p*-value = 0.048) were negatively correlated with MCP-1 in the whole sample (Table 3). Curiously, the levels of IGFBP-3 were significantly correlated with MCP-1 levels after treatment (BD1) (r_s_ (10) = −0.639, *p*-value = 0.047; Table 3). In the case of MIP-1β, only the levels of IGFBP-7 were significantly correlated in the whole sample (r_s_ (40) = 0.313, *p*-value = 0.049; Table 4). No other significant correlation was found between IGF proteins and TNF-α in any group (Table 4).

**TABLE 4:** Spearman’s correlation coefficient was the main correlation coefficient used.

Proteins	Groups	MCP-1 (pg/mL)		MIP-1β (pg/mL)		TNF-α (pg/mL)	
		r	*p*	r	*p*	r	*p*
IGF-2 (ng/mL)	All	0.043	0.791	0.188	0.244	−0.063	0.701
	C	0.176	0.458	0.402	0.079	−0.298	0.202
	BD	−0.183	0.439	0.006	0.98	0.171^P^	0.471
	BD0	0.224	0.533	0.262^P^	0.464	0.240^P^	0.504
	BD1	−0.234^P^	0.516	−0.067	0.855	0.203^P^	0.575
IGFBP-1 (ng/mL)	All	0.463**	**0.003**	0.148	0.362	0.043	0.793
	C	0.572**	**0.008**	0.010^P^	0.965	0.045^P^	0.850
	BD	0.377	0.101	0.129	0.587	−0.153^P^	0.519
	BD0	0.139	0.701	0.212^P^	0.556	−0.174^P^	0.631
	BD1	0.247^P^	0.492	0.127	0.726	−0.052^P^	0.866
IGFBP-3 (ng/mL)	All	−0.444**	**0.004**	−0.127	0.435	−0.186	0.251
	C	−0.310	0.184	0.267^P^	0.256	−0.221^P^	0.349
	BD	−0.087	0.715	0.131	0.582	0.028^P^	0.907
	BD0	0.176	0.627	0.209^P^	0.563	−0.176^P^	0.626
	BD1	−0.639* ^**P**^	**0.047**	0.273	0.446	−0.121^P^	0.740
IGFBP-5 (ng/mL)	All	−0.314*	**0.048**	−0.272	0.089	−0.290	0.069
	C	−0.177	0.456	−0.058^P^	0.809	−0.374^P^	0.104
	BD	0.275	0.240	−0.023	0.925	−0.169^P^	0.477
	BD0	0.152	0.676	0.414^P^	0.234	−0.023^P^	0.949
	BD1	−0.459^P^	0.182	−0.115	0.751	−0.300^P^	0.400
IGFBP-7 (ng/mL)	All	0.124	0.447	0.313*	**0.049**	−0.120	0.462
	C	0.197	0.405	0.312^P^	0.181	−0.386^P^	0.093
	BD	−0.059	0.806	0.260	0.268	−0.056	0.816
	BD0	−0.055	0.881	0.273	0.446	0.479	0.162
	BD1	−0.310^P^	0.384	0.018	0.960	−0.074^P^	0.839

r, coefficient values range from −1 to 1. ^P^Pearson’s correlation coefficient was used when both distributions adjust to a normal distribution (Shapiro–Wilk test; *p*-value ˃ 0.05). All (*n* = 40), C + BD groups; C (*n* = 20), control group; BD (*n* = 20), bipolar disorder; BD0 (*n* = 10), bipolar disorder time 0 (before treatment at hospital admission in the longitudinal subgroup); BD1 (*n* = 10), bipolar disorder time 1 (after treatment at hospital admission in the longitudinal subgroup); IGF, insulin-like growth factor; IGFBP, insulin-like growth factor-binding protein; MCP-1, monocyte chemoattractant protein 1; MIP-1β, macrophage inflammatory protein 1 beta; TNF-α, tumor necrosis factor alpha. * *p*-value <0.05 and ** *p*-value <0.01.

### 3.5 Correlation between IGF proteins, inflammatory markers, and the YMRS

No significant correlation was found between any IGF protein and inflammatory marker measured and the YMRS in bipolar patients either at hospital admission (BD0) or after treatment (BD1) (Table 5). Intriguingly, when we calculated the difference in the YMRS before and after treatment, we found that there was a significant correlation with the difference in IGF-2 levels (r_p_ (10) = 0.727, *p*-value = 0.017; Supplementary Table S5) and also the IGF-2 percentage reduction with treatment (r_p_ (10) = 0.687, *p*-value = 0.028; Supplementary Table S5).

**TABLE 5:** Pearson’s correlation coefficient was the main correlation coefficient used.

Protein	YMRS			
	BD0 (*n* = 10)		BD1 (*n* = 10)	
	r	*p*	r	*p*
IGF-2 (ng/mL)	−0.503	0.139	0.067	0.854
IGFBP-1 (ng/mL)	−0.506	0.135	−0.123	0.734
IGFBP-3 (ng/mL)	0.180	0.620	0.485	0.155
IGFBP-5 (ng/mL)	0.078	0.830	0.239	0.506
IGFBP-7 (ng/mL)	−0.503^s^	0.424	0.204	0.571
MCP-1 (pg/mL)	0.231^s^	0.521	−0.328	0.354
MIP-1β (pg/mL)	−0.064	0.860	−0.322^s^	0.364
TNF-α (pg/mL)	0.036	0.921	−0.083	0.820

r, coefficient values range from −1 to 1. ^s^Spearman’s correlation coefficient was used when at least one of the distributions did not adjust to a normal distribution (Shapiro–Wilk test; *p*-value <0.05). BD0, bipolar disorder time 0 (before treatment at hospital admission in longitudinal subgroup); BD1, bipolar disorder time 1 (after treatment at hospital admission in longitudinal subgroup); IGF, insulin-like growth factor; IGFBP, insulin-like growth factor-binding protein; MCP-1, monocyte chemoattractant protein 1; MIP-1β, macrophage inflammatory protein 1 beta; TNF-α, tumor necrosis factor alpha. YMRS, Young Mania Rating Scale.

## 4 Discussion

### 4.1 The peripheral IGF system in bipolar disorder

The main novelty in this study is the exploration of some members of the IGF signaling system in BD patients who had suffered a manic episode and the evaluation of their alterations in response to treatment conditions. To the best of our knowledge, this is the first time that IGF-2, IGFBP-1, IGFBP-3, IGFBP-5, and IGFBP-7 proteins have been peripherally measured in BD patients. On the contrary, in the last decades, IGF-1 has been the most studied IGF member in the context of BD.

First, genetic association studies found that IGF-1 was a potential candidate gene for BD susceptibility (Pereira et al., 2011). IGF-1 gene expression was significantly decreased in the subependymal zone in BD patients (Weissleder et al., 2021). Other studies evaluated the plasma levels of IGF-1 as a potential biomarker in BD patients (Palomino et al., 2013; Kim et al., 2013; Liu et al., 2014; da Silva et al., 2017; Tuncel et al., 2020). One study conducted in Spain found that there was no significant difference in IGF-1 levels between first-psychotic episode BD patients and controls neither at baseline nor after 1, 6, and 12 months of treatment (Palomino et al., 2013). Although we could not study first-episode BD patients, we had previously showed that both IGF-2 and IGFBP-7 were significantly increased in first-episode SZ Spanish patients (Fernández-Pereira et al., 2022). Curiously, during the first psychotic episode, the levels of IGF-1 in SZ patients were significantly higher than those in BD patients but not in controls (Palomino et al., 2013). Bearing this in mind, it would be interesting to study whether there is an imbalance in IGF-2 levels at the onset of bipolar disorder.

Later on, higher IGF-1 serum levels were found in BDI patients during a manic episode (Liu et al., 2014; Tuncel et al., 2020) and during euthymia (da Silva et al., 2017), meaning that this increase might not be just a state manic-dependent aspect but a more general trait condition in BD patients. Two different meta-analyses based on the above-mentioned studies concluded that IGF-1 peripheral levels are significantly increased in BD patients. Curiously, no correlation was found between IGF-1 levels and age, body mass index (BMI), age of onset, and gender (Tu et al., 2016; Chen MH et al., 2020). Despite the fact that most studies were performed on BD patients suffering from a manic episode, no correlation between the YMRS and IGF-1 peripheral levels were found either at baseline (Palomino et al., 2013; Kim et al., 2013; Liu et al., 2014; da Silva et al., 2017) or after treatment (Kim et al., 2013; Palomino et al., 2013). On the same line, we did not find any significant correlation between any IGF protein either before or after treatment and the YMRS. It has to be mentioned that we did find a significant positive correlation between both the difference and the percentage reduction before and after treatment in IGF-2 levels and the difference in the YMRS. This might suggest that the higher the difference or the percentage reduction in IGF-2 levels, the higher the difference or response to treatment. Nonetheless, the standard used to estimate treatment responsiveness is the percentage reduction of the YMRS (Chengappa et al., 2003; Berwaerts et al., 2011) but not the difference.

Regarding treatment type, IGF-1 levels were not different between BD patients treated either with lithium or valproic acid (Tuncel et al., 2020) or other drugs (Kim et al., 2013). Notably, it has been shown in a lymphoblastoid cell line that the IGF-1 gene was overexpressed in BD-I patients who were lithium responders in contrast to both controls and non-responders (Squassina et al., 2013). IGF-2 has some overall functions different from IGF-1 since, e.g., IGF-2 does not mediate growth hormone effects (Miller et al., 2022). We believe that IGF-2 requires further study as well in the context of human psychiatry since both IGF-1 and IGF-2 share common signaling pathways triggering IGF-1R that play key roles in neurodevelopment (Harris and Westwood, 2012). In addition, IGF-2 exhibits IGF-1R-independent actions due to its binding to IGF-2R, which has been recently suggested as responsible for mediating its cognitive and neuroprotective effects (Alberini, 2023). Nonetheless, we did not find any previous study that evaluated IGF-2 in BD patients. Our results suggest that there are neither significant differences in IGF-2 levels between controls and BD patients nor a statistical alteration with treatment.

On the other hand, the IGFBPs are binding proteins that mediate IGF actions (Song et al., 2021), but they also have IGF-independent actions (Mohan and Baylink, 2002). However, we have measured IGF-2 only in BD patients with a manic episode, and since we have recently found that IGF-2 were significantly increased in MDD patients (Fernández-Pereira et al., 2023), we believe that it would be interesting to explore IGF-2 levels in BD patients suffering a depressive episode. In some cases, IGFBPs can serve as a portable storage for IGF ligands by increasing their peripheral half-life, modulating their binding with their respective cell surface receptors (Duan and Xu, 2005) and impacting their downstream effects. Interestingly, we found that both IGFBP-3 and IGFBP-5 were significantly downregulated in BD patients compared to controls. Both IGFBP-3 and IGFBP-5 were the only IGF proteins studied that were decreased in BD patients. Since IGFBP-3 transports almost 80% of IGF ligands peripherally (Ranke, 2015), its mechanism of regulation could be different from those of the other IGFBPs, meaning that a decrease in IGFBP-3 may be balanced by an increase in IGF free forms, which could increase their downstream effects. In the case of IGFBP-5, its mechanism of regulation could be a consequence of IGFBP-3 downregulation since we found that levels of both proteins were significantly and strongly correlated in almost all groups independently of the psychiatric or treatment condition. In terms of binding proteins, previous studies were mainly focused on IGFBP-2 (Milanesi et al., 2018). Specifically, it was found that IGFBP-2 protein serum levels were significantly reduced in BD patients compared not only to matched controls but also other mental conditions such as depression (Milanesi et al., 2018). Interestingly, Milanesi et al. (2018) found that there was no statistical difference between BD patients who were using lithium or valproate treatment as mood stabilizers. Moreover, a significant decrease in *IGFBP-2* mRNA expression was measured in the *post-mortem* frontal cortex of BD patients, with a specific statistical decrease among patients who were receiving lithium treatment (Bezchlibnyk et al., 2007). One considerable aspect of potential biomarkers in psychiatry is that their changes can recreate the treatment response and so are used with subjective scales to evaluate the severity of symptomatology in clinics. In this work, we found that only IGFBP-1 was significantly reduced after treatment. Interestingly, lithium chloride dosages significantly reduced in a dose-dependent manner the expression of IGFBP-1 mRNA and protein secretion in a *in vitro* cell model (Lewitt et al., 2001). However, the tendency of the other IGF proteins was to be reduced after treatment conditions, although the difference was not statistically significant. The expression of IGFBP-2 mRNA and protein levels changed in a dose-dependent manner in response to therapeutic concentrations of lithium in a rat culture of cortical neurons (Bezchlibnyk et al., 2006). However, these changes might be specific to cortical neurons and not detectable in plasma since we did not find any statistical difference in IGFBP-7 levels between BD patients and controls either before or after treatment.

### 4.2 Altered peripheral inflammatory markers in bipolar disorder

The innate immune dysfunction has been considerably evaluated in the pathophysiology of bipolar disorder as well (Rosenblat, 2019), and many investigations have evaluated whether the cytokine alterations are a trait marker, postulating BD as a low-grade inflammatory disease, or a more state-dependent marker, meaning that inflammatory peaks are a result of acute mood episodes but might not be present during euthymia or treatment response (Munkholm et al., 2018). To begin with, TNF-α has been, by far, one of the most studied cytokines in the last decades. As proven in animal models, TNF-α is also produced by fat cells (Hotamisligil et al., 1993), and so, it has been postulated that the drugs used in our study that produce weight gain, such as olanzapine (Kraus et al., 1999) or amitriptyline (Hinze-Selch et al., 2000), would activate the TNF-α system and increase the plasma levels of TNF-α as a consequence. A decade ago, it was proved that TNF-α levels were non-different between both overweight BDI patients and controls (Barbosa et al., 2012a), which makes the Body Mass Index (BMI) a substantial factor to consider when evaluating peripheral inflammation in BD patients. Nonetheless, in our case, TNF-α peripheral levels decreased after treatment in responders despite the reduction not being significant. Other studies found that treatment responders who had suffered a manic episode experienced a significant reduction in TNF-α levels (Li et al., 2015). In addition, lithium has also been demonstrated to upregulate TNF-α levels (Himmerich et al., 2005) even in euthymic patients compared to both drug-free patients and controls (Guloksuz et al., 2010). Moreover, patients who showed a better lithium response had significantly decreased TNF-α levels (Guloksuz et al., 2012; Remlinger-Molenda et al., 2012). Conversely, other studies showed that a subset of patients who were following treatment with antipsychotics or lithium did not show statistical differences in TNF-α levels compared to controls (Zazula et al., 2022), as in our cohort, made up of patients under antipsychotic treatment.

Some studies have also measured soluble TNF receptors (TNFRs), which are found in 60 kDa and 80 kDa forms (Teixeira et al., 2015). In contrast to TNF-α, research on soluble TNF receptors has obtained more contradictory results (Millett et al., 2020). Two meta-analyses found increased 60 kDa sTNFR levels in BD patients (Modabbernia et al., 2013; Munkholm et al., 2018). In some cases, increased 60 kDa sTNFR levels in the manic phase were found in contrast to controls (O'Brien et al., 2006; Ortiz-Domínguez et al., 2007; Brietzke et al., 2009). Nevertheless, another study showed increased 60 kDa sTNFR and 80 kDa sTNFR levels also during the euthymic phase (Doganavsargil-Baysal et al., 2013). Conversely, higher 80 kDa sTNFR levels were postulated as a state marker of the depressive phase, and both sTNFR 60 and sTNFR 80 kDa levels were associated with the severity of depression but not mania (Siwek et al., 2017b). The most recent meta-analysis made in the field revealed that 60 kDa sTNFR levels are significantly higher in BD patients (Goh et al., 2023). To some extent, the alteration in the levels of the soluble TNF receptors indicates an alteration in the TNF-α signaling pathway (Doğanavşargil Baysal et al., 2019).

BD manic patients showed significantly higher TNF-α levels than controls at admission (Kim et al., 2007; Junior et al., 2023; Polat et al., 2023), without receiving pharmacotherapy 2 (Pandey et al., 2015) or 3 weeks before protein measurement (Ortiz-Domínguez et al., 2007), when following stable treatment (O'Brien et al., 2006; Kim et al., 2007; Kapczinski et al., 2011; Luo et al., 2016; Wang et al., 2016; Hoseth et al., 2017; Koga et al., 2019; Bavaresco et al., 2020; Zazula et al., 2022; Lu et al., 2023; Gong et al., 2022), and in both early (Skibinska et al., 2022) and late BD stages (Kauer-Sant Anna et al., 2009; Tatay-Manteiga et al., 2017), or significantly reduced levels in euthymic late patients but not in early BD patients (Panizzutti et al., 2015). In other cases, no difference was found between controls and BD patients who were under medication (Barbosa et al., 2011; Palacio et al., 2016; Doğanavşargil Baysal et al., 2019; Garés-Caballer et al., 2022; Jiang et al., 2022) with valproic acid monotherapy (Chou et al., 2016) or at baseline when specified that BD patients had a manic episode (Li et al., 2015; Jacoby et al., 2016) or were in their first episode (Chen MH et al., 2020). During euthymia, TNF-α has also been unaltered compared to controls (Kunz et al., 2011; Barbosa et al., 2012a; Doganavsargil-Baysal et al., 2013; Wieck et al., 2014; Jacoby et al., 2016; Doğanavşargil Baysal et al., 2019; Millett et al., 2020; Vares et al., 2020). Nonetheless, in some cases, BD patients who suffered a manic episode showed increased levels compared with euthymic patients (Fiedorowicz et al., 2015) or controls (Polat et al., 2023) when being drug-free. Nonetheless, after 6 weeks of treatment, these levels were significantly reduced (Uyanik et al., 2015). Conversely, few studies found significantly reduced TNF-α levels during acute manic episodes or remission in BD1 patients (Pantović-Stefanović et al., 2018) or euthymia (Su et al., 2023). Moreover, as it was in our case, no study has found any correlation between manic symptomatology through measures with the YMRS and TNF-α levels in BD patients (O'Brien et al., 2006; Kim et al., 2007; Barbosa et al., 2011; Doganavsargil-Baysal et al., 2013; Luo et al., 2016; Pantović-Stefanović et al., 2018; van den Ameele et al., 2017). Taking into account the most recent meta-analysis in the field, TNF-α levels are significantly higher during both manic and depressive episodes but not during euthymia periods (Solmi et al., 2021). In our case, we found a trend of a low-grade inflammatory state in BD patients when suffering from a manic episode that was reduced after treatment, although not statistically significant, which could be due to the small sample size or the different treatments that the patients were following by the time response was achieved.

Conversely, a smaller number of studies have evaluated MCP-1 in the context of BD. Some studies have found no significant difference in MCP-1 between BD patients during euthymia and controls (Brietzke et al., 2009; Barbosa et al., 2012b; Jakobsson et al., 2015), while others found that MCP-1 is significantly increased (Drexhage et al., 2011; Bai et al., 2014). On the other hand, studies have compared MCP-1 between mood states among different patients. One study found that MCP-1 was altered during depression or mania compared to euthymia but not when the whole sample considered as BD was compared to controls (Fiedorowicz et al., 2015). Others found that there was no significant difference between mood states and euthymia (Bai et al., 2014; Barbosa et al., 2012b). Noticeably, MCP-1 levels seemed to be significantly increased in non-responsive depressive BD patients who were under lithium treatment when compared to responders (Benedetti et al., 2017). This could be somehow in line with our findings since our patients who responded to the pharmacological treatment showed a general reduction in MCP-1 levels, although it did not reach statistical significance. As a main conclusion, a meta-analysis conducted in 2020 that included eight studies determined that MCP-1 levels are significantly increased in BD patients compared to controls, but after subgroup analysis, this difference was reduced to depressive mood states (Misiak et al., 2020). Shortly after, a study evaluated MCP-1 as a potential marker in treatment response in depressive BD patients, and no significant difference was found in MCP-1 between BD non-responsive depressive patients and controls (Edberg et al., 2020). Interestingly, the levels of MCP-1 were significantly reduced in non-responders after 4 and 8 weeks of treatment when compared to responders. Nonetheless, in the same patients, the levels of MCP-1 did not change over time (Edberg et al., 2020). Recently, the levels of MCP-1 were found significantly reduced in both moderate and severe depressive BD but not in manic BD patients (Wu et al., 2023). To some extent, more studies may be needed in the field to shed light on whether peripheral monocyte activation through MCP-1 levels is increased in BD patients and whether this alteration is related to acute mood states rather than BD itself. In our case, we did find that MCP-1 was the only inflammatory marker elevated during manic episodes that reached statistical meaning. Regarding controlling factors, some studies found that MCP-1 levels are positively correlated with age but not with the medication in use, such as lithium, valproic acid, or antipsychotics (Barbosa et al., 2012b). Conversely, we did not find any statistical correlation between age and MCP-1 levels either in the control or the BD group. This could be due to either the smaller sample size in our study or because we only included patients suffering from a manic episode, whereas in their research, half of the patients were euthymic (Barbosa et al., 2012b). In addition, one study determined that MCP-1 levels were positively correlated with the YMRS score (Bai et al., 2014), which might suggest that the higher the severity of the symptoms during mania, the higher the peripheral inflammatory state. However, this was not detectable in our study either before or after pharmacological treatment.

On the other hand, we did find only a few studies that related MIP-1β plasma or serum levels in BD. Initially, the gene expression of MIP-1β was found to be non-significant in the peripheral blood of mononuclear cells from BD chronic patients compared to controls (Brambilla et al., 2014). Moreover, there is a potential correlation between polygenic risk scores (PRSs) for the first psychosis episodes in BD patients with higher MIP-1β serum levels (Maj et al., 2020), which indicates that this chemokine might play a role in the inflammatory alteration and onset of illness vulnerability. In addition, recent research showed that in BD patients, adverse childhood experiences are associated with higher peripheral levels of MIP-1β and TNF-α but not MCP-1 (Poletti et al., 2022). In the present study, we did not find any differences in MIP-1β levels between BD patients and controls or a change in response to treatment. This could be due to the fact that we included chronic patients, and these alterations have been observed at the onset of the psychiatric disorder. As a whole, this inflammatory environment, along with alterations in neurotrophic factors, may predispose a toxic system that could lead to prejudicial alterations in the BBB structure and function in BD patients (Zhao et al., 2022).

### 4.3 Limitations and future perspectives

In our study, we faced the following limitations: we did not have access to some potential confounding parameters, such as glucose, cholesterol, triglycerides, albumin, and BMI in both patients and controls. Inconveniently, we could not compare treatment types. However, this study was conducted on a clinical daily basis, where patients have been prescribed multiple pharmacological treatments according to expert judgment. The relatively small sample size in our study could be considered as another limiting factor. On the other hand, whether IGF peripheral changes reflect alterations at the brain level is still a matter of study. To some extent, future research with bigger sample sizes will help understand the pharmacological action in the IGF signaling system, along with the peripheral inflammatory state in BD patients that could shed some light on whether this family of proteins might protect against neuroinflammation under this mental condition and whether we can use them as potential diagnostic, prognostic, or monitoring biomarkers.

## Data Availability

The original contributions presented in the study are included in the article/Supplementary Material; further inquiries can be directed to the corresponding author.
