# Supplementary material for: Plasma IGFBP-3 and IGFBP-5 levels are decreased during acute manic episodes in bipolar disorder patients
Source: Front Pharmacol. 2024 Apr 24;15:1384198. doi: 10.3389/fphar.2024.1384198 (PMC11076695; doi:10.3389/fphar.2024.1384198)
Supplement: Supplementary file 1 [file Table1.doc]

Supplementary Material

# Supplementary Data

**Supplementary Table 1.** Correlation between Insulin-like growth factor (IGF) proteins and inflammatory markers with age in all groups.

**Supplementary Table 2.** Insulin-like growth factor (IGF) and inflammatory markers differences in male and female in all groups.

**Supplementary Table 3.** Correlation among Insulin-like growth factor (IGF) proteins in all groups.

**Supplementary Table 4.** Correlation among inflammatory markers in all groups.

**Supplementary Table 5.** Correlation between differences and percentage reductions after pharmacological treatment in the levels of Insulin-like growth factor (IGF) proteins and inflammatory markers with the Young Mania Rating Scale (YMRS) differences and percentage reductions.

# Supplementary Figures and Tables

| Proteins | Groups | Age (years) | | Proteins | Groups | Age (years) | |
| --- | --- | --- | --- | --- | --- | --- | --- |
|  |  | r | p |  |  | r | p |
| IGF-2 (ng/ml) | All | 0.151 | 0.352 | IGFBP-1 (ng/ml) | All | 0.227 | 0.159 |
|  | HC | 0.374^s^ | 0.104 |  | HC | 0.444 | 0.051 |
|  | BD | 0.035 | 0.885 |  | BD | 0.031 | 0.898 |
|  | BD0 | 0.144 | 0.692 |  | BD0 | 0.498 | 0.143 |
|  | BD1 | -0.036 | 0.922 |  | BD1 | 0.039 | 0.915 |
| IGFBP-3 (ng/ml) | All | -0.153^s^ | 0.347 | IGFBP-5 (ng/ml) | All | -0.146 | 0.370 |
|  | HC | 0.124 | 0.603 |  | HC | -0.083 | 0.727 |
|  | BD | -0.170 | 0.473 |  | BD | -0.040 | 0.868 |
|  | BD0 | -0.472 | 0.168 |  | BD0 | -0.352 | 0.319 |
|  | BD1 | -0.271 | 0.450 |  | BD1 | -0.083 | 0.821 |
| IGFBP-7 (ng/ml) | All | **0.319** | **0.045** | MCP-1 (pg/ml) | All | 0.227^s^ | 0.159 |
|  | HC | **0.506^s^** | **0.023** |  | HC | 0.427^s^ | 0.061 |
|  | BD | 0.135^s^ | 0.570 |  | BD | 0.114^s^ | 0.632 |
|  | BD0 | -0.232^s^ | 0.519 |  | BD0 | 0.323^s^ | 0.362 |
|  | BD1 | -0.208 | 0.565 |  | BD1 | 0.176 | 0.627 |
| MIP-1β (pg/ml) | All | **0.316** | **0.047** | TNF-α (pg/ml) | All | 0.011^s^ | 0.945 |
|  | HC | 0.018 | 0.942 |  | HC | 0.082^s^ | 0.730 |
|  | BD | **0.470** | **0.036** |  | BD | -0.040 | 0.867 |
|  | BD0 | 0.320 | 0.368 |  | BD0 | -0.159 | 0.661 |
|  | BD1 | 0.451^s^ | 0.191 |  | BD1 | -0.591 | 0.072 |

Supplementary Table 1. Pearson’s correlation coefficient was the main coefficient employed. r: coefficient values range from -1 to 1. ^s^ Spearman’s correlation coefficient was used when at least one of both distributions did not adjust to a normal distribution (Shapiro-Wilk test; p-value < 0.05). All (n = 40): HC + BD groups; HC (n = 20): healthy control; BD (n = 20): bipolar disorder; BD0 (n = 10): bipolar disorder time 0 (before treatment at hospital admission in longitudinal subgroup); BD1 (n = 10): bipolar disorder time 1 (after treatment at hospital admission in longitudinal subgroup); IGF: Insulin-like growth factor; IGFBP: Insulin-like growth factor binding protein; MCP-1: Monocyte chemoattractant protein 1; MIP-1β: Macrophage inflammatory protein 1 beta; TNF-α: Tumor necrosis factor alpha. p-value < 0.05 indicates statistical significance.

| Proteins | Groups | Gender (F/M) | | p |
| --- | --- | --- | --- | --- |
|  |  | F | M |  |
| IGF-2 (ng/ml) | All | 124.35 ± 54.81 | 125.43 ± 62.28 | 0.954 |
|  | HC | 121.85 ± 47.12 | 107.21 ± 55.35 | 0.532 |
|  | BD | 126.86 ± 64.09 | 143.65 ± 66.24 | 0.572 |
|  | BD0 | 170.83 ± 55.89 | 155.11 ± 48.59 | 0.648 |
|  | BD1 | 163.01 ± 57.76 | 174.80 ± 49.91 | 0.739 |
| IGFBP-1 (ng/ml) | All | 7.49 ± 4.55 | 9.59 ± 4.07 | 0.052^1^ |
|  | HC | 6.38 ± 3.59 | 9.96 ± 5.31 | 0.089^1^ |
|  | BD | 8.61 ± 5.30 | 9.23 ± 2.54 | 0.742 |
|  | BD0 | 7,01 ± 4.54 | 6.91 ± 2.15 | 0.964 |
|  | BD1 | 10.68 ± 6.49 | 9.56 ± 2.44 | 0.728 |
| IGFBP-3 (ng/ml) | All | 389.28 ± 134.18 | 349.15 ± 121.64 | 0.328 |
|  | HC | 504.06 ± 79.12 | 444.59 ± 77.81 | 0.107 |
|  | BD | 274.50 ± 49.75 | 253.71 ± 70.28 | 0.455 |
|  | BD0 | 268.83 ± 42.90 | 231.03 ± 28.29 | 0.139 |
|  | BD1 | 286.39 ± 34.76 | 229.26 ± 13.08 | **0.008^1^** |
| IGFBP-5 (ng/ml) | All | 60.98 ± 28.69 | 54.82 ± 28.55 | 0.501 |
|  | HC | 85.36 ± 13.04 | 79.55 ± 16.48 | 0.684^1^ |
|  | BD | 36.59 ± 15.69 | 30.10 ± 9.52 | 0.278 |
|  | BD0 | 42.39 ± 5.35 | 37.23 ± 4.81 | 0.147 |
|  | BD1 | 48.60 ± 6.08 | 37.59 ± 5.42 | **0.016** |
| IGFBP-7 (ng/ml) | All | 121.63 ± 126.08 | 91.66 ± 66.00 | 0.678^1^ |
|  | HC | 75.56 ± 28.29 | 64.62 ± 29.92 | 0.412 |
|  | BD | 167.71 ± 167.46 | 118.71 ± 81.72 | 0.853^1^ |
|  | BD0 | 133.94 ± 49.30 | 175.78 ± 121.94 | 0.841^1^ |
|  | BD1 | 274.66 ± 180.78 | 135.51 ± 70.93 | 0.310^1^ |
| MCP-1 (pg/ml) | All | 87.22 ± 43.11 | 94.43± 70.98 | 0.968^1^ |
|  | HC | 65.72 ± 22.31 | 78.91 ± 31.06 | 0.209 |
|  | BD | 108.72 ± 48.98 | 109.95 ± 95.58 | 0.481^1^ |
|  | BD0 | 115.07 ± 42.34 | 82.22 ± 20.15 | 0.310^1^ |
|  | BD1 | 67.44 ± 32.06 | 90.38 ± 47.74 | 0.310^1^ |
| MIP-1β (pg/ml) | All | 62.89 ± 34.59 | 61.22 ± 90.13 | 0.068^1^ |
|  | HC | 48.69 ± 26.14 | 38.49 ± 20.98 | 0.348 |
|  | BD | 77.10 ± 37.34 | 83.95 ± 124.74 | 0.123^1^ |
|  | BD0 | 83.61 ± 31.27 | 44.80 ± 51.73 | 0.189 |
|  | BD1 | 71.95 ± 71.52 | 34.58 ± 29.76 | 0.312 |
| TNF-α (pg/ml) | All | 33.84 ± 12.45 | 48.23 ± 26.98 | 0.072^1^ |
|  | HC | 32.58 ± 12.52 | 39.43± 21.21 | 0.391 |
|  | BD | 35.10 ± 12.92 | 57.03 ± 30.25 | 0.063^1^ |
|  | BD0 | 126.86 ± 64.10 | 143.65 ± 66.25 | 0.573 |
|  | BD1 | 27.91 ± 9.22 | 41.95 ± 32.86 | 0.690^1^ |

Supplementary Table 2. Parametric Student’s T test was the main statistical test employed. ^1^ Non-parametric Mann-Whitney’s test was used if at least one of both distributions did not adjust to a normal distribution (Shapiro-Wilk test; p-value < 0.05). F: Female. M: Male. All (F/M; 20/20): HC + BD; HC (F/M; 10/10): healthy control; BD (F/M; 10/10): bipolar disorder; BD0 (F/M; 5/5): bipolar disorder time 0 (before treatment at hospital admission in longitudinal subgroup); BD1 (F/M; 5/5): bipolar disorder time 1 (after treatment at hospital admission in longitudinal subgroup); IGF: Insulin-like growth factor; IGFBP: Insulin-like growth factor binding protein. MCP-1: Monocyte chemoattractant protein 1; MIP-1β: Macrophage inflammatory protein 1 beta; TNF-α: Tumor necrosis factor alpha. p-value < 0.05 indicates statistical significance.

| IGF Proteins | Groups | IGFBP-1 (ng/ml) | | IGFBP-3 (ng/ml) | | IGFBP-5 (ng/ml) | | IGFBP-7 (ng/ml) | |
| --- | --- | --- | --- | --- | --- | --- | --- | --- | --- |
|  |  | r | p | r | p | r | p | r | p |
| IGF-2 (ng/ml) | All | 0.172 | 0.287 | 0.067^S^ | 0.681 | 0.003 | 0.984 | **0.814^S^** | **<0.0001** |
|  | HC | 0.136^S^ | 0.567 | 0.414^S^ | 0.070 | 0.317^S^ | 0.173 | **0.836^S^** | **<0.0001** |
|  | BD | 0.244 | 0.300 | 0.157 | 0.509 | 0.262 | 0.264 | **0.767^S^** | **<0.0001** |
|  | BD0 | 0.087 | 0.812 | 0.052 | 0.886 | 0.159 | 0.662 | **0.903^S^** | **<0.0001** |
|  | BD1 | -0.082 | 0.823 | 0.153 | 0.673 | -0.071 | 0.846 | **0.737** | **0.015** |
| IGFBP-1 (ng/ml) | All |  |  | -0.214^S^ | 0.184 | -0.093 | 0.570 | **0.322^S^** | **0.043** |
|  | HC |  |  | -0.329 | 0.157 | -0.338 | 0.146 | 0.227 | 0.335 |
|  | BD |  |  | -0.090 | 0.704 | 0.367 | 0.112 | 0.355^S^ | 0.125 |
|  | BD0 |  |  | -0.521 | 0.122 | -0.460 | 0.181 | 0.139^S^ | 0.701 |
|  | BD1 |  |  | 0.088 | 0.810 | 0.283 | 0.428 | -0.061 | 0.867 |
| IGFBP-3 (ng/ml) | All |  |  |  |  | **0.810^S^** | **<0.0001** | 0.021^S^ | 0.898 |
|  | HC |  |  |  |  | **0.653** | **0.002** | **0.459** | **0.042** |
|  | BD |  |  |  |  | -0.082 | 0.732 | 0.385^S^ | 0.094 |
|  | BD0 |  |  |  |  | **0.953** | **<0.0001** | 0.042^S^ | 0.907 |
|  | BD1 |  |  |  |  | **0.811** | **0.004** | **0.652** | **0.041** |
| IGFBP-5 (ng/ml) | All |  |  |  |  |  |  | -0.047^S^ | 0.773 |
|  | HC |  |  |  |  |  |  | 0.360 | 0.119 |
|  | BD |  |  |  |  |  |  | 0.371^S^ | 0.107 |
|  | BD0 |  |  |  |  |  |  | 0.152^S^ | 0.676 |
|  | BD1 |  |  |  |  |  |  | 0.418 | 0.229 |

Supplementary Table 3. Pearson’s correlation coefficient was the main coefficient employed. r: coefficient values range from -1 to 1. ^S^ Spearman’s correlation coefficient was used when at least one of both distributions did not adjust to a normal distribution (Shapiro-Wilk test; p-value < 0.05). All (n = 40): HC + BD groups; HC (n = 20): healthy control; BD (n = 20): bipolar disorder; BD0 (n = 10): bipolar disorder time 0 (before treatment at hospital admission in longitudinal subgroup); BD1 (n = 10): bipolar disorder time 1 (after treatment at hospital admission in longitudinal subgroup); IGF: Insulin-like growth factor; IGFBP: Insulin-like growth factor binding protein. p-value < 0.05 indicates statistical significance.

| Infl. Markers | Groups | MIP-1β (pg/ml) | | TNF-α (pg/ml) | |
| --- | --- | --- | --- | --- | --- |
|  |  | r | p | r | p |
| MCP-1 (pg/ml) | All | **0.496** | **0.001** | 0.287 | 0.072 |
|  | HC | 0.429 | 0.059 | 0.388 | 0.091 |
|  | BD | **0.556** | **0.011** | 0.053 | 0.823 |
|  | BD0 | **0.758** | **0.011** | 0.042 | 0.907 |
|  | BD1 | 0.261 | 0.467 | -0.255^P^ | 0.478 |
| MIP-1β (pg/ml) | All |  |  | 0.013 | 0.935 |
|  | HC |  |  | -0.049^P^ | 0.836 |
|  | BD |  |  | -0.056 | 0.813 |
|  | BD0 |  |  | 0.007^P^ | 0.985 |
|  | BD1 |  |  | -0.006 | 0.987 |

Supplementary Table 4. Spearman’s correlation coefficient was the main coefficient employed. r: coefficient values range from -1 to 1. ^P^ Pearson’s correlation coefficient was used when both distributions adjust to a normal distribution (Shapiro-Wilk test; p-value ˃ 0.05). All (n = 40): HC + BD groups; HC (n = 20): healthy control; BD (n = 20): bipolar disorder; BD0 (n = 10): bipolar disorder time 0 (before treatment at hospital admission in longitudinal subgroup); BD1 (n = 10): bipolar disorder time 1 (after treatment at hospital admission in longitudinal subgroup); MCP-1: Monocyte chemoattractant protein 1; MIP-1β: Macrophage inflammatory protein 1 beta; TNF-α: Tumor necrosis factor alpha. p-value < 0.05 indicates statistical significance.

| Proteins | | YMRS (dif) | | YMRS (% red) | |
| --- | --- | --- | --- | --- | --- |
|  |  | r | p | r | p |
| IGF-2 (ng/ml) | Dif | **0.727** | **0.017** | 0.254 | 0.478 |
|  | %red | **0.687** | **0.028** | 0.282 | 0.430 |
| IGFBP-1 (ng/ml) | Dif | 0.589 | 0.073 | 0.302 | 0.397 |
|  | %red | 0.588 | 0.074 | 0.190 | 0.599 |
| IGFBP-3 (ng/ml) | Dif | 0.379 | 0.281 | 0.424 | 0.222 |
|  | %red | 0.309 | 0.384 | 0.392 | 0.263 |
| IGFBP-5 (ng/ml) | Dif | -0.067^S^ | 0.854 | 0.055^S^ | 0.881 |
|  | %red | -0.122^S^ | 0.738 | 0.105^S^ | 0.777 |
| IGFBP-7 (ng/ml) | Dif | 0.187 | 0.605 | 0.001 | 0.999 |
|  | %red | 0.309 | 0.386 | 0.209 | 0.562 |
| MCP-1 (pg/ml) | Dif | 0.419 | 0.228 | 0.390 | 0.265 |
|  | %red | 0.480^S^ | 0.160 | 0.139^S^ | 0.701 |
| MIP-1β (pg/ml) | Dif | 0.305 | 0.391 | -0.179 | 0.621 |
|  | %red | 0.389^S^ | 0.266 | -0.006^S^ | 0.987 |
| TNF-α (pg/ml) | Dif | -0.065 | 0.859 | -0.112 | 0.759 |
|  | %red | -0.193 | 0.593 | -0.310 | 0.384 |

Supplementary Table 5. Pearson’s correlation coefficient was the main coefficient employed. r: coefficient values range from -1 to 1. ^S^ Spearman’s correlation coefficient was used when at least one of both distributions did not adjust to a normal distribution (Shapiro-Wilk test; p-value < 0.05). Dif: difference in values in BD patients after (BD1) and before (BD0) treatment (BD1-BD0). % red: percentage reduction in values in BD patients after (BD1) and before (BD0) patients ((BD1-BD0)/BD1*100). IGF: Insulin-like growth factor; IGFBP: Insulin-like growth factor binding protein. MCP-1: Monocyte chemoattractant protein 1; MIP-1β: Macrophage inflammatory protein 1 beta; TNF-α: Tumor necrosis factor alpha. YMRS: Young Manic Rating Scale. p-value < 0.05 indicates statistical significance.
